# Chicago Gynæcological Society; Meeting December 17th

**Published:** 1887-02

**Authors:** 

**Affiliations:** 2330 Indiana avenue


### TRANSACTIONS OF THE CHICAGO GYNÆCOLOGICAL SOCIETY.

*Regular Meeting, Friday, December 17th, 1886.*

*I.—Presentation of Specimens.*

*II.*—ETHERIDGE. *A Case of Anterior Vaginal Enterocele.*

*III.*—SÄNGER. *Ætiology, Pathology, and Classification of Salpingitis.*

*IV.*—WESTON. *Metritis.*

The PRESIDENT, CHARLES WARRINGTON EARLE, M.D., in the Chair.

### I. PRESENTATION OF SPECIMENS.

DR. HENRY T. BYFORD exhibited the following specimens:

#### 1. PROLIFERATING OVARIAN CYSTOMA.

This specimen, removed three weeks ago by Dr. William H. Byford, at the Woman's Hospital, is interesting in being a large ovarian cystoma, composed of an immense number of small cysts. Extremely formidable in appearance—resembling a malignant growth—it has pursued an exceedingly benign course. Adhesions were few and easily severed. The subsequent recovery was typical, one dose of morphine constituting the entire medication.

2. PAROVARIAN CYST AND APPENDAGES, COMPLICATED BY A UTERINE FIBROID.

This cyst, which was about the size of the head of a child two years old, developed between the layers of the broad ligament in a direction away from the uterus, lifting up the Fallopian tube, ovary and infundibulo-pelvic ligament. The tube is straightened and hypertrophied, admitting the finger for an inch into the fimbriated extremity, and the infundibulo-pelvic ligament is separated from its ordinary pelvic wall attachment by a larger portion of the tumor, over and under which the peritoneum passes to form, with the ovarian ligament and uterine end of the Fallopian tube, the pedicle. Had there been no pedicle, or had it been desirable to leave the ovaries, the inner coating of the cyst could have been taken out of the serous investment in about two minutes, without violence or hemorrhage. You will observe that I separate them now almost as easily as if they were wet pieces of linen lying in apposition. The ovary is slightly hypertrophied. On account of the presence of a uterine fibroid tumor, the size of an orange, the other healthy ovary and tube were removed. Three transparent cysts, about the size of beans, and feeling as hard as bone or wood are, as you see, hanging by long pedicles from the meso-salpinx. The ovarian artery runs over the tumor and under the tube, is elongated and four or five times its normal thickness. Here is also the left tube. It is congested, but neither it nor the artery is appreciably enlarged. The ovary is large but not pathological.

3. OVARIAN CYST IN THE BROAD LIGAMENT CONTAINING THE DEGENERATED OVARY.

This tumor, which I removed by enucleation and which was the size of a pregnant uterus in the sixth month, is interesting by comparison with the other, and also as illustrating the dangers of tapping. It evidently developed from the surface of the ovary between the layers of the broad ligament towards the uterus. The remainder of the ovary has developed into a multilocular cystoma the size and shape of a large orange, and projects into the large cyst. The firmness of the adhesions of this inner coat of the tumor to its peritoneal covering, as compared with the smaller cyst just shown, suggests that small tumors are more easily enucleated than large ones, and constitutes an argument in favor of earlier operations. The horn of the uterus with the Fallopian tube was hypertrophied and drawn up over the anterior surface of the tumor, so that when exposed during the operation it looked like a pregnant uterus with a large vein running over it, diagonally across the incision. Had the tumor been tapped in the ordinary place, trouble must have resulted, and the operation which, after the stripping off of this hypertrophied and congested uterine tissue from the tumor, was easily and rapidly completed, would have been complicated. The uterus in a few weeks was apparently normal in size, position and mobility. It is interesting to note that the tumor was mistaken for advanced pregnancy, about a year ago, by one of our most prominent general practitioners.

*4.* SUPPURATING OVARIAN ADENOMA, WITH UTERUS.—AUTOPSY.

This tumor, which was examined for me by Dr. F. S. Johnson, is an ovarian adenoma of the same histological nature as the first specimen shown, but presents a striking contrast by malignant course and small size, as the whole was scarcely larger than a child's head. An ovarian tumor was removed from the opposite side by Dr. William H. Byford several years ago. As this was not a malignant growth, although practically pursuing the course of one, an attempt was also made by him to remove this, or at least cure the discharging abscess that surrounded it, about five months before the patient's death, but it was, from the nature of the case, only partially successful. In attempting its removal after death, I was almost equally unsuccessful, for it was surrounded and intermingled with pus cavities and intestinal loops, and had destroyed and occupied the place of all the pelvic tissues except the rectum, bladder and uterus with the right round ligament which you see hanging to it. The cul-de-sac was full of it, the posterior uterine surface inseparable from it, the right broad ligament supplanted by it, and the contents of the left broad ligament an agglomerated mass of inflammatory tissue. A blue walled abscess about the size of, and extending along the course of, the ascending colon, had secured an outlet just below the border of the ribs. Another abscess opening just below and to the right of the umbilicus gave exit to pus. feces and a semi-transparent, jelly-like ovarian fluid. The right ureter was dilated and hypertrophied. The pelvis of the kidney must also have been dilated, although that being left to be examined last,* was finally forgotten. The lower three feet of the ileum were compressed and atrophied to the size of a small lead pencil. Just above it ran a fibrous tube of the usual size of the ileum, straight from the cæcum to the lower opening in the skin. The uterus, which previous to the removal of the first tumor, had developed two healthy children, was of rather small size, in a normal position and healthy, excepting a moderate laceration of the cervix and a slight degree of injection of the mucous membrane about the os.

5. FIBRO-SARCOMA OF THE LEFT HORN OF THE UTERUS, LUNGS, PLEURA, PERICARDIUM, RECTUM, TRANSVERSE AND DESCENDING COLON AND ABDOMINAL PARIETES.

About a quart of serum was found in the right pleural cavity. Several round, fibro-sarcomatous tumors, the size of nuts, and several indefinite spots of contracted fibrous tissue, were found in the lungs, suspending their expansibility except over a few small areas. The two which I hold in my hand were torn from the lungs; one from the center of the apex and the other from the pleura at the base. A fibro-sarcoma the size of a goose's egg which is, as you see, still attached to the left pleura, was also attached to the pericardium. The heart weighed 8¾ ounces, was drawn to the left by the contracted lung (which had but little fluid about it) and contained an ante-mortem clot. The liver was enlarged and mottled. The stomach lay entirely to the left side, in front of the compressed and atrophied spleen, with the pyloric orifice suspended vertically under the cardiac orifice, and not reaching as far to the right as to the median line. The transverse and descending meso-colon was strung with these fibro-sarcomatous masses, the size of hazel nuts, and the rectum covered with the same of a little larger size. A few were also found on the peritoneal surface of the abdominal parietes. The uterine tumor weighed twenty ounces, and, like all of the specimens, cannot be distinguished from fibroid or fibrous tumors by the eye, except over a limited area upon the anterior surface, where it has softened and degenerated into the round celled variety. The

* The autopsy was performed in the midnight before the burial, and in a small town, in a cold room and without conveniences.

fact that the patient was twice tapped by physicians for ovarian cystoma makes it seem probable to me that this change has been produced by the introduction of the trocar, and that the little tumors on the abdominal parietes were due to the adhesions forming afterwards. The fluid obtained then was probably peritoneal which, to the amount of about eight pints, had again accumulated. It was dark green in color. Abdominal enlargement was noticed several years ago. An operation had been advised two years ago. Dr. F. S. Johnson examined the specimens, with the result mentioned, and but for an attack of sickness would have reported upon them in person.

DR. W. W. JAGGARD exhibited the following specimens:

1. UNILOCULAR CYST OF RIGHT OVARY, THE REMAINING OVARIAN TISSUE SHOWING CORPUS LUTEUM OF MENSTRUATION.

He had recently removed the tumor, and the patient had made an excellent recovery. Menstruation ceased three days before the operation. The specimen was interesting, though not exceptional, in showing the persistence of functional activity in such an extensively diseased organ. The left ovary was normal, and was not removed.

2. A PLACENTA, SHOWING VELAMENTOUS INSERTION OF THE UMBILICAL CORD, AND REMAINS OF AN EXTENSIVE HÆMORRHAGE INTO THE PARENCHYMA OF THE ORGAN.

The term *insertio velamentosa* means a separation of the three vessels of the cord before they reach the placenta. The vessels pursue a straggling course between chorion and amnion, for a variable distance, and each one reaches the placental margin by itself.

According to B. Schultze (*Jenaische Z. f. med. u Naturw*, 1867 Heft. 2 and 3) the origin of velamentous insertion of the umbilical cord is as follows: The allantois carries the fœtal vessels to the periphery of the egg, entirely irrespective of the future placental site. Indeed, it is comparatively seldom that the future placental site is immediately reached. The vessels penetrate all the chorionic villi indiscriminately. These vessels are subsequently obliterated in the chorionic villi, not destined to form the placenta, and vascular connection only remains with that portion of the chorionic villi, corresponding to the *decidua serotina*. With the further growth of the egg, under normal conditions,—it makes no difference what point in the egg periphery the allantois originally touched,—as the amniotic sheath forms around the rudimentary cord, the fœtus performs a movement of rotation, so that the vessels pursue a straight course to the placenta. Now this movement of rotation may be hindered, and the formation of a complete sheath prevented by anomalous adhesions. The hindrance is adhesions of the umbilical vesicle, the ductus, or the vessels to the amnion or chorion. In such cases, if the growth of the ductus does not keep equal pace with that of the amnion, a complete sheath cannot be formed, and the amnions forsake the vessels before they reach the placental edge. Velamentous insertion of the cord can occur at the pole of the egg opposite to the placenta or at any intermediate point. The anomaly is observed most frequently in cases of multiple pregnancy.

Velamentous insertion of the umbilical cord seldom leads directly to interference with the development of the fœtus. In labor, however, the vessels may be compressed between the presenting part and the parturient passage, and the child may be asphyxiated, or, with the rupture of the bag of waters, a vessel may be torn and the child's life endangered by loss of blood. In general terms, the clinical picture bears a great resemblance to *placenta prævia.*

In the specimen presented, the cord is inserted into the chorion about five centimeters from the placental edge. The anomaly was not recognized until near the conclusion of the first stage of labor. A large vessel was torn at the same time with the spontaneous rupture of the bag of waters, and the life of the child was seriously jeopardized by the loss of blood.

The child, though probably mature in point of age, was small and feeble; weight, 2,700 grams, length 47 centimeters. The failure in development could be ascribed to the large hæmatoma and numerous hæmorrhagic infarctions in the *placenta fœtalis.* The mother of the child was threatened with abortion, and suffered from uterine hæmorrhage when she was in the sixth month of pregnancy. The child, at the time of presentation of specimen, was living.

THE PRESIDENT exhibited the ileum, removed from a child 22 months old, who died of typhoid fever, in which the characteristic lesions of the disease could be seen.

The child had continued fever for five weeks, the temperature reaching 105° F. part of the time, rose-colored spots in the third week, intestinal hæmorrhage, tympanites, and delirium.

The case is remarkable on account of the age of the patient, as many authorities deny the existence of genuine typhoid in children.

Stewart (1841) describes infantile remittent fever in which the bowels are sometimes relaxed, occasional delirium, followed by a low, quiet state. Evidently he did not recognize typhoid as occurring in children.

Condie (1853) says, "It is much more than probable that typhoid will be found to be a much more frequent disease among children than has heretofore been supposed."

Bedford (1856) does not speak of typhoid.

Hillier (1868) says, "Typhoid is not infrequent in children. Many cases of infantile and gastric fever belong to this class."

Meigs and Pepper (1870) say, "Typhoid fever has been observed during the first year of life, but is rare under the age of two years. It is comparatively infrequent between the ages of three and eight years, and attains its maximum of frequency in childhood between the ages of eight and eleven years."

Tanner (1871) believes that all those varieties of typhoid which have hitherto been described under the names of simple, continued and remittent fever are but different degrees of one and the same disease, typhoid.

Eustace Smith (1884) says, "Enteric fever is common in children."

Steiner (1871) says of typhoid, "This is common among children. In the Prague Hospital, out of 80,245 patients, 1,180 had typhoid."

Vogel (1870) says, "Abdominal typhus is much more frequent than is commonly supposed."

I think J. Lewis Smith did not mention typhoid in his first edition, but in his sixth (1886) he describes it fully, believing that it is not infrequent in children and presents peculiarities not found in adults, hence his chapter of eleven pages.

From the above quotations it seems that foreign authors recognize it as moderately frequent and writers in this country who, a quarter of a century ago, either did not speak of the disease as occurring among children or gave the subject very slight mention, are now describing it as more common, even among quite young children.

In all probability, as our malarial diseases become rare, typhoid fever increases in frequency.

II.—DR. J. H. ETHERIDGE made the following remarks upon

A CASE OF ANTERIOR VAGINAL ENTEROCELE

with exhibition of patient.

I have a rare case that I would like to exhibit to the Society, one of anterior vaginal enterocele. I have had a few of my medical friends examine it, and they all concur in saying that they have never seen a case like it. The patient is 19 or 20 years of age and has one child, 11 months old. When she was about six months gone in pregnancy, she "jumped the rope" one day and after that felt something come down through the vagina. She went to full term and had a normal labor. Whenever she strains or lifts, the enterocele comes down, presses the vulva apart and comes out between the thighs. On examination I find quite a large opening in the roof of the vagina. The edges of the ring can be very easily outlined with the finger, and when the hernia is down the finger in the vagina is at once attracted by a pendant mass, and by pressing it a little one can determine that it is filled with gas. The opening comes down to the left of the uterus, anterior to the broad ligament posterior and to the left of the bladder.

A Fowler pessary has been fitted which seems to answer the purpose of complete retention, and as long as the patient can avoid an operation my advice is not to have one. The uterus is in good position. She made a good recovery from her confinement and is nursing her child, and seems to be in a perfectly physiological condition. The question of operative procedure for a radical cure is a very serious one for this patient. Laparotomy is full of difficulty in attempting to close the hernial opening from within, is of a most unsatisfactory possibility in its outcome, and is followed by the very great risk to life which attends all laparotomies. Any operation through the vaginal tract will be attended by such a lack of certainty in results as to cause me to hesitate in essaying it. Until it is found impossible to retain the intestine in its proper place with a pessary, I have advised the patient to avoid submitting to any surgical proceedure. The patient is present, and each gentleman desiring to do so can make the examination and verify what I have said.

DR. PHILIP ADOLPHUS: The case just presented for examination by Dr. Etheridge is a typical case of vaginal hernia, the intestine passing between the loosely attached connective tissue which unites the bladder and anterior wall of the vagina, in its upper half. This mode of descent is much more infrequent than a hernia into the *cul-de-sac* of Douglas. In the case here presented the hernia is reducible in the upright position, contains intestine only, and has for its covering the peritoneum and vaginal wall. What can be done for this patient's relief? These herniæ seldom become strangulated. During labor, however, besides being an obstacle to prompt delivery, they are liable to contusion and strangulation. No retentive apparatus is worthy of trial, for all distend the vagina and ultimately increase the evil. What surgical procedure should be attempted? The text-books to which I have access do not suggest anything. Is the hernia to be closed by way of abdominal section or *per vaginam*? I think the closure *per vaginam* is preferable, and I suggest the following procedure, adapted from Stoltz's operation for cystocele: "The patient being placed in Simon's position with the perineum retracted, the hernia is to be reduced and kept in place by means of armed probangs. An incision to be made over the tumor, the tissues divided until the ring of the hernia is exposed. This ring is to be surrounded by a running ligature of very heavy cat-gut, and then closely approximated, tied, and the ends cut off short, or, if thought preferable, interrupted cat-gut sutures may be introduced to effect the same purpose. This completes the first step of the operation. Then remove a piece of the vagina larger than the protruded tumor, over the region of the hernia. The wound may be closed by running a circular single ligature of carbolized silk in and out, about an eighth of an inch from the margin of the wound, all around it, the end of the ligature being brought out close to the place where it was first inserted. The two ends are then drawn tight and tied, leaving a puckered opening into which a little drainage tube may be inserted. During the introduction and the tightening of the ligature the intestine may be held back by armed probangs. It is easier to work in this region with the scissors than the knife. Great care should be practiced in operating in this neighborhood not to wound the uterus and the peritoneum, which can be avoided by elevating the mucous membrane as it is removed.

Now a few words in regard to the abdominal section for reducible vaginal enterocele. After having opened the abdominal cavity and withdrawn the prolapsed portion of the intestine, will it be easier to close the hernial aperture than *per vaginam*? I think not. The peritoneum and cellular tissue beneath it (abundantly supplied with lymphatics and blood-vessels, the parametric tissue of Virchow and Spiegelberg) will have to be incised, the bladder and upper portion of the vagina separated, the redundant tissue of the anterior wall of the vagina drawn up and excised, then stitched aud replaced; the peritoneum also closed below as well as externally in the abdominal parietes. Will this render the site of the hernial protrusion stronger? Will it be a safer and more efficient operation than the first? I think not.

DR. H. T. BYFORD: Dr. Etheridge has shown us one of those rare and interesting cases of anterior vaginal enterocele. The protrusion is through the parametrium in front and to the right of the cervix, the entire uterus being pushed backward, and the bladder forward. The left ureter can be felt passing from the trigone in front of the tumor downward and to the left of it. The broad ligament is pushed backward, and the round ligament outward toward her left side. The parts to the left of the median line seem only slightly to participate in the general displacement. The remedy for this condition, of course, must be to get the displaced organs and tissues back into the place now occupied by the intestine. I think Dr. Etheridge was wise in rejecting abdominal section as a remedy, for no advantage could be derived from it that would compensate for the risk involved. An operation from the vagina that would be justifiably performed upon a girl of her age could not be expected to be permanently successful, as the parts could not be properly replaced, and vaginal support could only be given. The treatment by pessary, already adopted in this case, is better than either of these procedures, because it acts partly by replacing the organs, especially the uterus, and partly by providing a barrier to the descent of the enterocele. Its weakness is that it does not tend to strengthen the parts upon it, and that it weakens those under it. The most efficient and rational method of permanently replacing the parts, and thus curing the enterocele would be to perform Alexander's operation of shortening the round ligaments. This would draw the fundus forward over the bladder and thus replace the normal central barrier to the descent of the intestines. It would also draw the round ligament and upper portion of the broad ligament forward to form a lateral support to them. By turning back the base of the broad ligament it would tend to draw back into position the ureter (which is in close relation with its posterior edge), and at the same time the connective tissue through which the ureter runs and which is displaced forward with it. I know of no other method in which the parts could be brought back so nearly to their former relations. A pessary could now be worn until the parts had contracted and regained tonicity. If then the anterior vaginal wall remained redundant a circular piece should be excised in front of the cervix and by a stitch passed around it drawn together, along with the parametric tissue immediately above it.

DR. ETHERIDGE: If Alexander's operation was satisfactorily performed, what would be the effect upon the bladder and upon subsequent pregnancy?

DR. BYFORD: After the operation the fundus is not, or should not be, held down upon the bladder as firmly as in some cases of anteflexion, in which there are no bladder symptoms of any account. I have performed three operations. In one case the bladder symptoms were benefited, in the others there were none complained of, either before or afterward. As to pregnancy, already there is some experience to show that it does not interfere. The round ligaments are not elongated in pregnancy as much as would be supposed, since the uterus grows to a certain extent away from them. In many cases of anteflexion or anteversion the ligaments are shorter than after an Alexander operation, yet give rise to no serious trouble in pregnancy.

DR. ADOLPHUS: How could Alexander's operation be of benefit if it draws the fundus down and the cervix up, since the enterocele is between the bladder and the cervix?

DR. BYFORD: It draws the body of the uterus down under the intestines, displacing them upward. The cervix needs to be held upward and backward in order to secure a proper relation of parts.

### III.—ÆTIOLOGY, PATHOLOGY AND CLASSIFICATION OF SALPINGITIS.

The Secretary, Dr. Edward Warren Sawyer, read the following communication from Dr. M. Sänger, of Leipsic, in reply to a letter by Mr. Lawson Tait, read before the Society, May 28th, 1886.

Dr. Sänger's letter was translated by Dr. Ivo Bernauer, of the Cook County Hospital, and revised by Dr. Christian Fenger.

LEIPSIC, October 10th, 1886, Lindenstrasse 16.

TO DANIEL T. NELSON, M.D., PRESIDENT OF THE CHICAGO GYNÆCOLOGICAL SOCIETY.

*Dear Sir :* I have been personally attacked by Mr. Lawson Tait in a letter addressed to you ; as I desire that my reply should go by the same way, I take the liberty of requesting you to bring my letter to the notice of your Society and to have it assigned a place in the Transactions of the same. Nobody will dispute that up to the present time Mr. Lawson Tait, of all laparotomists, has had the best results, at all events in regard to ovariotomy and salpingo-oöphorectomy. His practical results have, however, raised his conceit to so high a degree that in pathological questions also he assumes a certain infallibility, which vents itself in numerous sallies and attacks upon others. The consequence of this is that just at present, Mr. Lawson Tait is being subjected to various energetic criticisms as by Bigelow, Schröder and others.

Now Mr. Lawson Tait has also shot one of his shafts at me. I feel very thankful toward Dr. Christian Fenger for having received it on my account. However I do not wish to allow the matter to rest there.

At the sessions of the Gynæcological Society of Chicago, held in February and March, the treatment of pelvic abscess by laparotomy was discussed in a highly instructive manner. Dr. Fenger in his excellent and exhaustive remarks said that "my statements regarding ætiology were the most complete" inasmuch as there must necessarily be as many forms of pelvic abscess as there are forms of disease of the Fallopian tubes. The latter were enumerated by Dr. Fenger according to the classification given by me in a paper read at the *Versammlung Deutscher Naturforscher* in Magdeburg. (See report, Archiv. f. Gynæk. Bd. XXV, pp. 126–33.) In this classification six different forms are recognized, and it is this distinction which Lawson Tait is pleased to style "absurd." Although Dr. Fenger reiterated his statement "that he regarded my classification as correct and complete and in accordance with the laws governing inflammatory processes in all organs of the body," yet I do not think that this defense of my position is sufficient. Considering the great influence Lawson Tait exercises upon the profession, I deem it my duty to refute him in every particular. I shall attempt to do so in a scientific manner, and I may thus hope that my reply will prove of general use and interest.

The pathological anatomy and the course of salpingitis can be understood only when we bear in mind the theories of infection. The whole sexual tract, from the ring of the hymen to the *ostium tubæ abdominale*, is open to the entrance of the external air and the germs suspended in it. Carriers of infection coming from the abdominal cavity and its contained organs may also enter at the *ostium tubæ abdominale*. Even microbes originally lodged in the external parts, in the vagina around the cervix, may, by way of the lymphatics, reach the peritoneal cavity, and thence gain entrance into the tubes.

The normal vaginal and uterine secretions at the age of puberty and the menstrual blood contain numerous non-pathogenous micro-organisms. Still greater numbers are found in the catarrhal secretions of the uterus in cases of endometritis as was demonstrated by Küstner. As to the normal tubal secretion and the tubal secretion in cases of *salpingitis catarrhalis*, consequent upon *endometritis catarrhalis*, no investigations have as yet been made to show whether or not they, likewise, contain non-pathogenous microbes. However, as the secretion of an endometritis contains microbes we may assume that if the inflammation is continued into the tubes, its secretion will here likewise contain the same. It has been clearly proven that pathogenous micro-organisms pass from the external parts to the tubes and the peritoneal cavity a fact which is doubted by no one, perhaps, except by Lawson Tait. These organisms have in part been accurately studied and it is well known that different kinds produce distinct forms of salpingitis, and secondarily pelveo-peritonitis. The fact was already established by Gurèin and Guerrier, that in making vaginal and intra-uterine injections air, and thus, also micro-organisms, might pass into the tubes (Physo-Salpinx). S. Hennig, in his book "*Krankheiten der Eileiter*," p. 52, surmises that, in cases of putrid endometritis and physometra, gases may escape from the tubes into the peritoneal cavity.

Our present knowledge of the above mentioned pathogenous micro-organisms will enable us to divide them into three groups:

GROUP I.—FORMS OF SALPINGITIS PRODUCED BY KNOWN SPECIFIC MICROBES.

1. *Salpingitis gonorrhœica*, produced by the gonococcus of Neisser.

2. *Salpingitis tuberculosa*, produced by the *bacillus tuberculosis* of Koch.

3. *Salpingitis actinomycotica*, produced by the *actinomyces bovis* of Bollinger.

1. *Salpingitis gonorrhœica* is the only specific, infectious form of salpingitis which is recognized as such by Lawson Tait, although he stops short of admitting that the gonococcus is the exciting agent. Without doubt the gonorrhœal is the form most frequently met with. This fact was clinically established as early as 1872 by Nöggerath, long before Neisser had discovered his gonococci or Lawson Tait had performed his first operations "for suppuration of the uterine appendages." In Germany, I myself was one of the first gynæcologists who at our meetings showed the frequency of gonorrhœal salpingitis, emphasized its causal connection with pelveo-peritonitis, and removed by operation the gravely implicated uterine adnexa. (Magdeburg, 1884, and Munich, 1886.) Gonorrhœal salpingitis is never followed by a destructive "suppuration" of the uterine appendages: it remains invariably a disease of the surfaces of the mucous and serous membranes. The pus formed by the specifically diseased mucous membrane gradually distends the tube; in one class of cases in which there is a great accumulation of free pus the tube is transformed into a large sac with thin walls; in another in which the wall of the tube, especially its muscular tissue, is hypertrophied to a greater extent, the tube becomes much thickened and rigid. In most cases, both conditions are found, the uterine portion of the tube is thickened, the abdominal end dilated. The serous surfaces of the tubes, the albuginea of the ovaries, the serosa of the peritoneum are attacked or become pus secreting surfaces only in cases in which gonorrhœal pus has escaped from the tubes and thus infected the above named structures. We may then have peri-salpingitis, peri-oöphoritis, peri-metritis, s. *pelveo-peritonitis purulenta gonorrhœica*. I do not believe that gonorrhœal pus ever penetrates the walls of the tubes and thus produces these diseases. But a specific gonorrhœal inflammation of the mucous membrane of the tube, with secretion of pus into the cavity of the latter, is accompanied by a non-specific inflammation of the entire tubal wall. This may also excite peri-salpingitis, peri-oöphoritis and so forth; the organs involved may become adherent to each other and displaced, but we never meet with a purulent exudate of the same nature as that found in the cavity of the tube itself. This also explaines why, in some instances, gonorrhœal disease of the uterine appendages is accompanied by severe and violent symptoms, frequently resembling those of a peritonitis following perforation, whereas in other instances it develops insidiously, scarcely manifesting any symptoms at all. In the former cases, gonorrhœal pus escapes through the *ostium abdominale* into the peritoneal cavity; in the latter the inflammation of the external surfaces of the adnexa is non-specific in character.

According to what I have just stated, I must necessarily regard the terms, "suppuration of the uterine appendices and peri-uterine s. pelvic abscess," as inaccurate, and from a general pathological point of view, as productive of confusion; we invariably find free pus in the tubes and peritoneal cavity or an inflammation of the adnexa, but never destructive suppuration of the tissues of the pelvic organs. In cases in which abscesses are discovered in the walls of the tubes, in the tissues of the *ligamenta lata* and in the ovaries these abscesses are, as I shall later on show, due to septic infection, but not to gonorrhœa. The latter disease produces suppuration only on surfaces.

I purposely enlarged somewhat on gonorrhœal salpingitis and its consequences, as this form presents a typical example of infectious salpingitis in general.

There is one more point to which I wish to call attention. Gonococci have not always been discovered in pus coming from the tubes in cases in which clinically there existed no doubt as to the gonorrhœal nature of the infection. The conditions under which the gonococci are destroyed, or prevented from further development, have not yet been ascertained; further investigation will also have to show whether, in cases in which gonococci are absent, there are not present other microbes belonging to one of the groups mentioned further on.

2. *Salpingitis tuberculosa.*—Alfred Hegar's lately published work, "*Enstehung, Diagnose und Chirurgische Behandlung der Genitaltuberculose des Weibes,*" relieves me of the necessity of entering more fully into the consideration of this form of salpingitis. Lawson Tait denies the existence of this form, or rather, he admits it, but only "for the third and contracting stage of pyosalpinx." This admission simply discloses his ignorance of the true nature of tuberculous infection. The pus in a case of purulent salpingitis, whether it be gonorrhœal or otherwise, may of course undergo caseation. This was called tuberculization before Koch's discovery of the *bacillus tuberculosis*; now it is termed coagulation-necrosis, according to Cohnheim-Weigert. It is this, which Lawson Tait confounds with the genuine infection by the bacillus of tuberculosis.

A pyosalpinx may remain in this third stage indefinitely: a tuberculous salpingitis will never result therefrom unless there be added a tuberculous infection.

3. *Salpingitis actinomycotica.*—This form is called by Lawson Tait "an equally ridiculous subdivision based on mere theory, not on fact." It seems to me before making such an unintelligible assertion it would have been his duty to inquire whether there really is no case on record to support me in including this form in my enumeration. In my paper, above mentioned, I named the author who had furnished this case; I will now accurately give my authority: Adolph Zemann, "*Über die Aktinomycose des Bauchfells und der Baucheingeweide beim Menschen, Medicin. Jahrbücher der K. K. Gesellsch, Ärzte in Wein,*" 1883, S. 477, Fall 4. The tubes in this case were dilated and filled with pus and clumps of the actinomyces, their walls were thickened and exhibited numerous granulations produced by the fungus. The fungus had migrated either from the vagina or from the intestines which were found extensively adherent to the tubes. What Lawson Tait does not know, has no existence for him. "Germanica sunt, non leguntur."

GROUP II.—FORMS OF SALPINGITIS DUE TO SPECIFIC MICROBES IDENTICAL WITH THOSE PRODUCING TRAUMATIC INFECTION.

*Salpingitis septica (pyæmica, ichorosa, purulenta, diphtheritica).*—The term *salpingitis septica*, is rather general and inaccurate; as when speaking of a pyosalpinx we simply mean that the tube contains pus, when employing the term *salpingitis septica* we merely indicate that the disease is due to infection by a septic virus. It is at the present time a matter of extreme difficulty to diagnose the nature of the pus and the nature of the infection presented to us in an individual case. Now, we certainly know that the microbes producing the different kinds of traumatic infection known clinically as septicæmia, pyæmia, diphtheria, phlegmon, erysipelas, may one and all invade the genital tract; we may hence infer the existence of an equal number of varieties of salpingitis, i. e. *salpingitis septica, pyæmica, diphtheritica, phlegmonosa, erysipelatosa.* In order to complete our scheme we should add *salpingitis putrida,* corresponding to putrid infection, whereby the difficulties are still further increased.

Notwithstanding the progress made in bacteriology, we have not yet succeeded in isolating and classifying the microbes which cause the clinically different forms of traumatic infection; consequently it is impossible to do this with regard to the different forms of *salpingitis septica.* However, the work done by Doléris, E. Fränkel, Lomer, A. H. Barbour, Nöggerath, Cushing, in the domain of puerperal infection has given us some positive results.

There are two points which are to be considred fundamental:

(1) The microbes of puerperal septicæmia are identical with those producing traumatic infection in general. During the puerperium after abortion, as well as after parturition at term, the genital tract is far more susceptible to infection, or the conditions are far more favorable to the spreading of infection, than at other times.

(2) As has been demonstrated by Ogston, Hueter and Rosenbach all suppuration is due to the action of microbes; several varieties of these, like the *streptococcus pyogenes* and *staphylococcus pyogenes,* have been closely studied, but it is known that they are not the only varieties which produce pus. As doubtless all of these carriers of infection may play a rôle in the production of salpingitis, we can readily see how complicated the question of infectious diseases of the tubes has become and how unscientific and untenable is the meaningless name of pyosalpinx. Yet there is a certain comfort in hoping that the matter may be somewhat simplified. Some of the forms of traumatic infection, for instance, sepsis, and diphtheria, pyæmia and phlegmon, are probably produced by identical micro-organisms, and the course of the disease may be modified by the nature of the tissue first attacked and by the manner in which the infection spreads, whether by the blood or lymph channels.

All of these infections, as is well known, have a double effect—a local one in the genital tract, and a constitutional one, which is brought about through the medium of the circulation, and which is seen not only in the system at large, but also in the localization of the infection in organs distant from the point of entrance of the micro-organism. When systemic effects are produced by the virus of putrid infection, the disease is called sapræmia; when by the virus of septic infection, septic toxæmia or ptomaine poisoning; when by that of purulent infection, pyæmia.

We thus meet with an essential difference between the diseases of the genital tract, produced by the microbes of Group I, and those of the traumatic infection. The above mentioned severe and acute constitutional symptoms are but slightly indicated, or may even be absent in the diseases of the first group, i. e. gonorrhœa, tuberculosis, actinomycosis, whose course is chronic; whereas in diseases of the second group the rapidity of their development and the malignity of their course may almost entirely obscure the local disturbance in the genital tract.

As is readily seen, the septic diseases of the tubes are not independent diseases; aside from the local disturbances co-existing in the uterus, vagina and external genitals, the whole circulatory system is usually affected. In this respect, as Lawson Tait rightly remarks, septic disease of the tubes is not a specific ailment; this, however, is of no importance in an enumeration of the varieties of salpingitis. An endometritis or a *colpitis gonorrhœica* may co-exist with a *salpingitis gonorrhœica*, and in the same way *salpingitis septica* may be accompanied by other diseases of the sexual organs, which are due to the same cause. There is, of course, no such thing as an affection of the tubes merely.

*Salpingitis septica*, co-existing with severe puerperal septicæmia or "lymphatic peritonitis" has never as yet, it is true, given the surgeon an opportunity to remove the principal focus of the disease by extirpation of the tubes. It is possible, however, that under certain circumstances such a procedure might be indicated. B. S. Schultze, "*s. Verhandl. d. gynækolog. Section d. Versammlung deutscher Naturforscher*," in Berlin, 1886, has recently succeeded in amputating a puerperal uterus, in a case in which it was impossible otherwise to remove the placenta, which had become the source of septic infection. Lately two cases came to my knowledge in which the tubes burst from overdistention with pus, whose nature, whether gonorrhœal or septic, was not ascertained. In both cases a general peritonitis resulted which proved fatal, in one on the fourth, in the the other on the twenty-first day after confinement. It is clear that in both these cases the salpingitis had existed before delivery. I shall afterwards relate a case of my own in which this certainly was the condition. Cases of this kind will be diagnosed more frequently and more readily as soon as our attention has been called to them, and we may then expect to hear of their treatment by operation. Cases of salpingitis, consequent upon traumatic infection in non-puerperal women, are, of course, of much more frequent occurrence. The carriers of infection may, for instance, be directly introduced into the tubes by means of an infected sound. The introduction of a septic instrument into the uterine cavity may be followed by a septic salpingitis. In cases in which we observe an exudative pelveo-peritonitis after the introduction of an infecting sound, after an intra-uterine injection or a *curettement* of the *mucosa uteri*, the infection almost always spreads to the pelvic peritoneum by way of the tubes and exceptionally only through the muscular walls of the uterus. The severe systemic disturbances, the diseases of the uterus and pelvic peritoneum, may gradually subside, whereas the tubal affection remains. A pyosalpinx has formed, the tubes are filled with pus, which can have been produced only by the action of one of the specific microbes of traumatic infection: perhaps by the *staphylococcus pyogenes*.

Two cases of my own may serve as illustrations. In one of them a physician had performed *abraiso mucosæ uteri* without antiseptic precautions, infection took place and a *pelveo-peritontis exudativa* followed, after the subsidence of which I could easily feel each tube thickened to the size of a thumb. At the operation both tubes were discovered to have thinned walls and to contain thick pus resembling that found in an abscess. The ovaries were small and enveloped in masses of very dense, connective tissue. I removed the tubes, but left the ovaries. The woman made a good recovery. The adnexa had been in a healthy condition before the mucous membrane of the uterus had been scraped. I have already published the other case ("*Verhandlungen der Gesellsch. f. Geburtsh*," *zu Leipzig*, 17 April, 1882; "*Centralb. f. Gynæk.*," 1882, p. 558); multipara of twenty-nine years; three spontaneous deliveries, at the fourth *placenta prævia*, and forceps applied before complete dilatation of the os. Puerperal fever. Recovery, but permanent pains in the right epigastrium. A short time after, renewed pregnancy, in the third month of which a prominent gynæcologist ascertained disease of the right uterine appendages; at full term rapid and spontaneous delivery. On the third and subsequent days of the puerperium, chills, high intermitting fever, icterus, in short the symptoms of acute pyæmia. Death on the thirtieth day. The autopsy revealed *salpingitis purulenta dextra*, and several abscesses in the right ovary and right broad ligament. The remainder of the genital organs, the adnexa on the left side normal. I explain the course which the disease took in this case in the following way: At the fourth parturition, septic infection and localization of the disease in the uterus and the appendages on the right side; subsidence of the grave constitutional disturbances and persistence of a pyosalpinx, probably due to *streptococcus pyogenes*, latency of pyosalpinx during subsequent pregnancy. After delivery increased absorption of pus, abscesses in the ovary and broad ligament, acute pyæmia and death. In a case of this kind a septic-infectious disease, originally extending over large portions of the sexual tract, is finally concentrated in the tubes. Why should not such a case, in which the tubes chiefly appear affected, present a "specific ailment" as well as does a case of *pyosalpinx gonorrhoica*, in which *ceteris paribus* we have the same limitation of the disease to the tube as a principal focus for further infection.

What I desire to prove by these somewhat extensive remarks is, briefly, as follows:

1. Numerous cases of *salpingitis purulenta* ("pyosalpinx") are due to traumatic infection, are septic forms of salpingitis.

2. There are as many forms of septic salpingitis as there are forms of traumatic infection, and of microbes producing the same.

There is, however, an additional reason why Lawson Tait denies septic salpingitis to be a specific ailment. As we see from his startling remarks in the *Medical News*, April 24th, 1886, he does not believe in sepsis at all, does not believe in infection, denies the principles on which the practice of modern surgery and obstetrics is based. He has been taught nothing by the researches of Semmelweis and Lister, Pasteur and Koch. And why? Because his own success in combating septic infection is to him proof of the non-existence of septic infection. We are accustomed to Lawson Tait's reckless statements. Without taking the trouble to refute them scientifically, I but wish to call to mind his assertion that menstruation does not depend upon the ovaries, but upon the tubes, that the mortality of the Cæsarean operation is still 99⅔ per cent. His denial of traumatic infection is a statement of the same kind.

I should like to hear Lawson Tait's answer to the following questions:

1. What disease, before the introduction of Listerism, killed thousands of patients who had received wounds or who had been operated on? What, according to his views, was the cause of death in his own cases, when he lost patients after operations, if not, as everybody else believes, septic infection?

2. What is puerperal fever?

Lawson Tait disputes the correctness of our teachings regarding infection, but he has failed to give us any other explanation of its phenomena.

GROUP III.—FORMS OF INFECTIOUS SALPINGITIS PRODUCED BY SPECIFIC, BUT AS YET UNKNOWN, MICROBES.

1. *Salpingitis syphilitica.*—This form has been described by Bouchard and Lépine (*Gazette Med. de Paris*, 1866, No. 41). Both tubes were swelled to the thickness of fingers, and contained three gummata of the size of hazel nuts. The description given by these authors of the tubal disease agrees fully with the changes brought about by syphilis in other organs. Of more recent authors, Gill Wylie expresses his opinion that tubal syphilis does occur ("Diseases of the Fallopian Tubes," etc., January 24th and February 7th, *The Medical Record*, 1885). He says that "syphilis may cause salpingitis, just as it does otitis or ozæna." He also calls attention to the fact that "endometritis in syphilitic subjects has a most obstinate character." Compared with the actual observation of Bouchard and Lépine, the clinical remarks of Gill Wylie are, of course, of theoretical value only.

Like Lawson Tait, I, myself, have never yet had occasion to observe an undoubted case of tubal syphilis. We should bear in mind, however, that our attention has been but little called to the anatomy and clinical forms of this disease. We are not justified in denying this form altogether, as we are not in a position to dispute the reliable authors who have testified to its existence. Others may have seen what we ourselves have never had occasion to witness. I desired to give a most complete enumeration of the forms of infectious salpingitis hitherto described; I could as little have omitted *salpingitis syphilitica* as I could have done *salpingitis actinomycotica*, of which also up to date but one authentic case has been observed.

2. Occasionally we find in young girls, who have never had intercourse with a man, tubes filled with pus and pelveo-peritonitis. This has been accounted for in various ways. It has been said that in these cases a serous catarrh is intensified and changed to a purulent inflammation; that the suppuration is due to catching cold at the menstrual period, or to a trauma. These cases have also been adduced as evidence to show "that tubal suppuration is not always of gonorrhœal origin." Yet also in these cases there is always an infection, and usually a gonorrhœal infection. I, myself, have seen a comparatively large number of girls of all ages, from infancy to puberty, who were infected with gonorrhœa. We know how easily the infecting germs are carried from one person to another; for instance, parents and children may use the same sponge or bath tub; the germs may adhere to fingers, linen, etc. The girls infected may further spread the disease in school, and so on.

Aside from the gonorrhœal, the tuberculous infection is to be mentioned as a cause of pyosalpinx in young girls. Just at present I am treating a girl of 17, who is suffering from a disease of both appendages, complicated with pelveo-peritonitis. The tubes can be distinctly felt, and are thickened and nodular. She has a hectic appearance, like that of a consumptive; her lungs, however, are normal. The father of the girl was, not long ago, operated on for tubercular orchitis. I hope that the operation will confirm my diagnosis of *salpingitis tubecrulosa.*

Undoubtedly the microbes of traumatic infection may also, after accidental lesions of the external genitals, get into the tubes of young girls; they may also enter from the peritoneal cavity. I know of a case, a girl of 16, in which an inflammatory disease of the right uterine appendages developed, consequent upon a retro-typhlitis.

My reason for again considering these forms of infectious salpingitis belonging to Groups I and II, in regard to their occurence in children is this: there are evidently other infections of the female genital tract, which have been observed in children but which have not as yet been recognized as special forms; consequently they are to be placed in Group III, in opposition to the forms of the two preceding groups. E. Fränkel, of Hamburg ("*Bericht über eine bei Kindern beobachteten Endemie infectiöser Kolpitis, Virch. Arch.*" February, 1885), and Johann C'seri, Budapest ("*Zur Ætiologie der*

*in fectioesen Vulvo-vaginitis bei Kindern,*" *Wiener Med. Wochenschrift*, 1885), describe an infectious disease of the vagina and vulva, the former believing it to be due to a special coccus, the latter to a coccus identical with Neisser's gonococcus.

Hennig (*Krankheiten der Eileiter*, page 67) in a girl of ten and Eppinger (*Prager Zeitschrift für Heilkunde*, 1882, page 36) in an adult witnessed a dysenteric inflammation extend to the mucous membrane of the genital tract; the disease in its new location assumed the appearance of a diphtheritic inflammation.

Suppuration is sometimes observed in the vaginæ of children suffering from helminthic disease. In these cases the suppuration is not caused by the irritation of the parasite especially the oxyuris, but by certain microbes carried into the vagina by the parasites. The nature of these microbes has not as yet been sufficiently investigated to allow of their classification.

It has long been known that certain infectious diseases, like typhoid fever, scarlatina, variola, cholera, may invade the genital tract. The local affections are probably caused by the same specific microbes which produce the typical constitutional disease. This, however, still remains to be proven.

At the close of this enumeration, I wish to repeat that in every case where the vagina or uterus is the seat of one of the diseases named, such disease may extend into the Fallopian tubes. I am well aware that I am standing on an unsafe scientific basis regarding my third group of forms of infectious salpingitis. For this very reason I deemed it advisable to group them together, thus keeping them apart from the better known forms; besides I desired to point out the object which should be kept in view in investigating this subject and in endeavoring to elucidate it still further. It is, finally, self-evident, that there are also mixed forms of salpingitis. Different varieties of micro-organisms may enter the tubes either simultaneously or successively. The forms most frequently found combined are the microbes of the gonorrhœal and tuberculous, and of the gonorrhœal and traumatic infection and those of the different varieties of the latter.

Every physician whose scientific standpoint is the same as mine will admit, that matters regarding salpingitis are immensely more complicated than Lawson Tait imagines, and that it is not sufficient to make an abdominal section, to remove the uterine appendages and to satisfy oneself with the diagnosis of pyosalpinx in case the tubes are found filled with pus; but that it is our duty, employing all the means furnished by modern science, to endeavor to make an accurate diagnosis of the form of disease affecting the uterine appendages before the operation, and afterwards to add to our clinical observation careful pathological and bacteriological examination of the specimen. This certainly is a higher standpoint than that occupied by Lawson Tait, to whom the removal of the uterine appendages is the chief thing, and who, neither before nor after the operation, concerns himself with the nature of the disease treated. He admits, himself, that in every fifth case he made an error in diagnosis. In a man like Lawson Tait, so great in his own estimation, it seems rather small to attempt to conceal his ignorance by resorting to insulting and scurrilous remarks in regard to German scientists. I advise Mr. Lawson Tait to learn German and to read the works of German gynæcologists; he may perhaps come to the conclusion that there is much which he might profitably study.

Here in Germany, Lawson Tait is held in high esteem, as he deserves to be, on account of his brilliant practical results. We have long since, however, ceased to regard as serious his theoretical utterances, which pretend to be scientific. The tone which he adopts in his polemic writings does not prevail with us in Germany, and it is certainly looked upon as undignified by every gentlemanly Englishman.

I am, sir, etc., DR. M. SAENGER,

*Privatdocent at the University of Leipsic, President of the Obstetrical Society of Leipsic.*

IV.—DR. EDWARD B. WESTON on presentation of a thesis, entitled,

METRITIS, read by the SECRETARY, was elected Fellow of the Society.

W. W. JAGGARD, M.D., *Editor.*

2330 Indiana avenue.

